# IP-10 detection in urine is associated with lung diseases

**DOI:** 10.1186/1471-2334-10-333

**Published:** 2010-11-22

**Authors:** Angela Cannas, Ludovica Calvo, Teresa Chiacchio, Gilda Cuzzi, Valentina Vanini, Francesco N Lauria, Luigia Pucci, Enrico Girardi, Delia Goletti

**Affiliations:** 1 Department of Epidemiology and Preclinical Research, L. Spallanzani National Institute for Infectious Diseases (INMI), Rome, Italy; 2 Translational Research Unit, Department of Epidemiology and Preclinical Research, INMI, Rome, Italy; 3 Pneumology Unit, Health Department, INMI, Rome, Italy; 4 Clinical Biochemistry and Pharmacology Laboratory, INMI, Rome, Italy

## Abstract

**Background:**

blood cytokines and chemokines have been proposed as biomarkers for tuberculosis (TB). Recently, some immune mediators found in the urine of patients with renal dysfunctions have also been suggested as potential biomarkers. Finding biomarkers for TB in urine would present several advantages over blood in terms of collection and safety. The objective of this study was to investigate the presence of cytokines and chemokines in the urine of patients with pulmonary TB at the time of diagnosis. In a subgroup, the evaluation was also performed during TB treatment and at therapy completion. Patients with lung diseases other than TB, and healthy subjects were also enrolled.

**Methods:**

urine samples from 138 individuals, after exclusion of renal dysfunctions, were collected during an 18 month-period. Among them, 58 received a diagnosis of pulmonary TB, 28 resulted having lung diseases other than TB, and 34 were healthy subjects. Moreover, 18 TB patients, 9 of whom were tested 2 months after AFB smear sputum reversion and 9 of whom were cured of TB were also included. Cytokines and chemokines in urine were evaluated using a Cytometric-Bead-Array-Flex-Set. IP-10 detection in 49 subjects was also carried out in parallel by using an Enzyme Linked ImmunoSorbent Assay (ELISA).

**Results:**

IFN-γ, TNF-α, IL-2, IL-8, MIP-1α, MIP-1β and RANTES were poorly detected in all urine samples. Conversely, IP-10 was consistently detected in urine and its level was significantly increased in patients with lung disease compared to healthy subjects (p < 0.001). Increased IP-10 levels were found in both pulmonary TB and lung diseases other than TB. Moreover lower IP-10 levels were found in cured-TB patients compared to the levels at the time of diagnosis, and this difference was close to significance (p = 0.06). Interestingly, we demonstrated a significant correlation between the data obtained by flow cytometry and ELISA (r^2 ^0.82, p < 0.0001).

**Conclusions:**

IP-10, in contrast to IFN-γ, TNF-α, IL-2, IL-8, MIP-1α, MIP-1β and RANTES, is detectable in the urine of patients with pulmonary diseases in the absence of renal dysfunctions. Moreover, the IP-10 level in cured-TB patients is comparable to that found in healthy subjects. More studies are needed to further investigate the clinical utility of these findings.

## Background

Tuberculosis is a leading cause of death worldwide, especially in low-resource settings, killing 1.8 million people each year [1]. Improved diagnostic tools that are more sensitive and easier to perform are needed for optimal identification and treatment of the disease [2].

Detection of the immune mediators interferon (IFN)-γ, tumor necrosis factor (TNF)-α, interleukin (IL)-2, IL-8, macrophage inflammatory protein (MIP)-1α, MIP-1β, RANTES and IFN-γ inducible protein (IP)-10 in blood have been suggested as potential biomarkers for TB [3-9]. Specifically, serum concentrations of IL-2, IL-6 and TNF-α, shown to be increased in patients with active TB [10-12], return to normal levels after treatment [13]. Similarly, IP-10, a CXC chemokine [14,15], has also been shown to be involved in the response to *Mycobacterium tuberculosis *infection and disease. Recent studies demonstrated that active tuberculosis (TB) is associated with increased IP-10 plasma levels when compared to controls [16], and that it is useful for monitoring therapy efficacy.

Recently, several immune mediators present in the peripheral circulation have been detected in the urine of patients with lupus nephritis [17], acute pyelonephritis during pregnancy [18] and in elderly subjects with urinary tract infections (UTI) [19], and are proposed as biomarkers for these kidney-related diseases.

Demonstrating that urine is a good biological sample for TB diagnosis would represent several advantages over blood. Collection of urine is non-invasive, does not present biological risks for the staff involved and does not require special equipment or highly specialized healthcare staff. More importantly, urine can be easily obtained in children. All these factors are highly relevant in poor resource settings.

It has been previously shown that the neopterin, an immune marker produced by human macrophages specifically on stimulation with IFN-γ [20], is increased in the urine of patients with several diseases as sarcoidosis [21], celiac disease [22], multiple sclerosis [23], transplants [24] and the acquired immune-deficiency syndrome (AIDS) [25]. In patients with active TB, urine neopterin has been demonstrated to be a useful parameter for measuring the degree of disease activity and the response to treatment [26-30].

However, to date (to our knowledge) there is no published evidence evaluating immune mediators as cytokines and chemokines, in the urine of TB patients.

Our study was designed to assess whether it is possible to detect those cytokines/chemokines known to be associated with TB in urine in order to find potential and useful clinical biomarkers for TB disease activity. An evaluation of these immune mediators was performed on a subgroup of patients during TB treatment and at therapy completion. Patients with lung diseases other than TB and healthy subjects were also enrolled.

## Methods

### Study participants

This study was approved by the Institutional Review Board at INMI. All study participants gave their written informed consent and were enrolled at the National Institute for Infectious Diseases (INMI), Rome, Italy from September 16^th^, 2008 until February 1^st^, 2010. In Italy the incidence of TB is 7 cases per 100,000 inhabitants. In Latium, the region where Rome is located, there are approximately 10 cases per 100,000 inhabitants, and about 60% of those are in immigrants [31].

Patients with suspected active pulmonary TB disease were prospectively and consecutively enrolled before starting therapy. Patients with past cases of TB were excluded. After registering the eligible subjects, three sputum samples were collected from each and all underwent radiological examinations. The collected sputum samples were processed, stained for acid fast bacilli (AFB) microscopy by Ziehl-Neelsen method and cultured in Lowenstein Jensen (BD Becton, Dickinson and Company, Milan, Italy) and in liquid BACTEC MGIT 960 (BD Becton, Dickinson and Company, Milan, Italy). The presence of *M. tuberculosis *in the positive culture samples was further confirmed by MTD Gen-probe based PCR (BioMérieux Inc., Marcy I'Etoile, France) method.

TB was defined as microbiologically confirmed if *M. tuberculosis *was isolated from sputum culture. Conversely, patients were classified as having "clinical TB" if the diagnosis was based on clinical and radiological criteria (after excluding other diseases), including appropriate response to anti-TB therapy (in terms of clinical recovery of the initial symptoms and healing of the radiological lung lesions).

Patients with pulmonary diseases other than TB, referred to as "lung diseases other than TB", had a final diagnosis based on microbiology and cytological tests, clinical signs, and successful treatment. Moreover 18 TB patients, 9 of whom were tested 2 months after acid-fast bacilli (ABF) smear reversion (mean time of therapy 71.25 ± 11.25 days) and 9 of whom were cured of TB (within 12 months of completion of therapy) were also included. As controls we included healthy laboratory staff.

Presence of UTI in the enrolled individuals was ruled out by evaluating bacteriuria, using an automated system (Aution Max, Menarini, Italy & Sysmex UF100, Dasit, Italy) and clinical criteria. Information regarding age, sex, ethnicity, BCG status and exposure to *M. tuberculosis *was collected through a structured questionnaire. Laboratory staff evaluating the presence of the immune factors was blinded to the status of the patients.

### Urine specimen collection and processing

Fifty ml of urine were collected from each individual in the study. Ethylenediaminetetraacetic acid (EDTA) [0.5 M EDTA-0.5 M Tris(hydroxymethyl)aminomethane hydrochloride (Tris-HCl), pH 8.5], was added to a final concentration of 40 mM within 30 min of collection, since the content and stability of nucleic acids in these samples was evaluated for other studies [32,33]. The urine specimens were then stored in 4 ml aliquots and stored at -80°C. Thawed urine samples were centrifuged at 3,000 rpm for 10 min at 4°C before analysis.

### Determination of chemokines and cytokines in urine

The quantitative determination of IFN-γ, TNF-α, IL-2, IL-8, MIP-1α, MIP-1β, RANTES and IP-10 urine concentrations was carried out using a human Cytometric Bead Array Flex Set Assays (CBA Flex Set; Becton Dickinson Biosciences, San Diego, CA), analyzed by flow cytometry (FACS Canto II, Becton Dickinson) according to the manufacturer's instructions. The majority of the urine samples were tested in duplicate wells. After the first evaluation, IP-10 (CXCL-10) was found to be the most interesting chemokine to evaluate. We used the same technology (according to the manufacturer's instructions) but only looked for IP-10. In a subgroup of subjects, IP-10 levels were also analyzed using Human CXCL10/IP-10 Quantikine ELISA (R&D Systems, Abingdon, UK) according to the manufacturer's instructions.

### Statistical analysis

The main outcome of the study was the evaluation of chemokine and cytokine production expressed as continuous (pg/ml) measures. Mean and standard deviation (SD) were calculated: an unpaired t-test was used for pair-wise comparisons and ANOVA was used to compare means among the various groups. Differences were considered significant at p values ≤ 0.05. SPSS v 14 for Windows (SPSS Italia Srl, Bologna, Italy) and Prism 5 software (Graphpad Software 5.0, San Diego, CA, USA) were used in the analysis.

## Results

### Characteristics of the population

Characteristics of the study participants are shown in Table 1. Eighty-six patients hospitalized with pulmonary symptoms suggestive of active TB were enrolled before starting therapy. Fifty-eight of these patients were later diagnosed with active pulmonary TB and 28 with "lung diseases other than TB". Forty-eight of the 58 (82.7%) TB patients resulted microbiologically confirmed, while 10 received a final clinical diagnosis. As controls, 34 healthy subjects were included. UTI was excluded by clinical evidence and by bacteriuria evaluation [bacteriuria levels of patients with pulmonary TB (mean: 1911, SD:1988), with "lung diseases other than TB" (mean: 1944, SD:1446) and healthy subjects (mean: 1346, SD:787). Significant differences between the groups were found in the categories of age, origin and BCG status. The subjects with "lung diseases other than TB" were older than the others (p = 0.003); the origins of those with TB were more heterogeneous compared to the others (p < 0.0001); and those with TB were more frequently BCG-vaccinated compared to the others (p < 0.0001) (Table 1)].

**Table 1:** Demographic and clinical characteristics of the subjects enrolled in the study.

	Total	Pulmonary TB	Lung disease other than TB	Healthy subjects	Pulmonary TB after 2 mo AFB smear reversion	Pulmonary TB Cured	p value
	N (%) 138 (100.0)	N (%) 58 (42.1)	N (%) 28 (20.3)	N (%) 34 (24.6)	N (%) 9 (6.5)	N (%) 9 (6.5)	
**Mean Age (SD)**	35.87 (12.06)	32.43 (10.63)	41.89 (14.54)	38.88 (8.06)	31.60 (6.92)	32.11 (19.18)	=0.003
**Female gender**	48 (34.8)	18 (31.0)	8 (28.6)	16 (47.1)	1 (11.1)	5 (55.6)	=0.137
**Origin**							< 0.0001
**Eastern Europe**	46 (33.3)	31 (53.4)	5 (17.9)	-	4 (44.4)	6 (66.7)	
**Western Europe**	49 (35.5)	10 (17.2)	4 (14.3)	32 (94.1)	1 (11.1)	2 (22.2)	
**Africa**	14 (101)	4 (6.9)	10 (35.7)	-	-	-	
**South America**	7 (5.1)	2 (3.4)	1 (3.6)	2 (5.9)	1 (11.1)	1 (11.1)	
**Asia**	22 (15.9)	11 (19.0)	8 (28.6)	-	3 (33.3)	-	
**BCG**							< 0.0001
**BCG-positive**	61 (44.2)	39 (67.2)	14 (50.0)	6 (17.6)	1 (11.1)	2 (22.2)	
**BCG-negative**	57 (41.3)	10 (17.2)	4 (14.3)	28 (82.4)	8 (88.9)	7 (77.8)	
**BCG-unknown**	19 (15.8)	9 (15.5)	10 (35.7)	-			
**Pulmonary TB** **microbiological diagnosis**	-	48 (82.8)	-	-	9 (100.0)	9 (100.0)	
**Pulmonary TB** **clinical diagnosis**	-	10 (17.2)	-	-			
**Pulmonary diseases** **other than TB**							
**Pneumonia**	-	-	16 (57.1)	-			
**Cancer**	-	-	2 (7.1)	-			
**Bronchitis**	-	-	3 (10.7)	-			
COPD*	-	-	6 (21.4)	-			
**Pleural effusion** **not TB related**	-	-	1 (3.6)	-			
**Mean Bacteriuria** **(SD)**	1770 (1632)	1911 (1988)	1944 (1446)	1346 (787)	685 (830)	2347 (1817)	=0.352
**HIV status**							=0.118
**HIV-positive**	4 (2.9)	1 (1.7)	2 (7.1)	-	1 (11.1)	-	
**HIV-negative**	126 (91.3)	55 (94.8)	23 (82.1)	34 (100.0)	5 (55.6)	9 (100.0)	
**HIV-unknown**	8 (5.8)	2 (3.4)	3 (10.7)	-	3 (33.3)	-	

TB: tuberculosis; HIV: human immunodeficiency virus; SD: standard deviation; BCG: Bacillus Calmètte et Guerin; COPD: chronic obstructive pulmonary diseases.

### Cytokine and chemokine analysis

The quantitative determination of IFN-γ, TNF-α, IL-2, IL-8, MIP-1α, MIP-1β, RANTES and IP-10 concentrations in urine was performed using a human CBA Flex Set, a flow cytometry application that allows for simultaneous quantification of multiple immune mediators.

All of the above -listed factors were first tested in a subgroup of 20 individuals and the levels of the immune mediators were almost undetectable. With the exception of IL-8 and MIP-1β, there were no significant differences between the two groups. Note: if the concentrations' means were below 5 pg/ml, no statistical analysis was performed (Table 2).

**Table 2:** Urinary levels of tested chemokines and cytokines in patients with pulmonary TB and healthy subjects.

	Pulmonary TB N. 10	Healthy subjects N. 10	p value
**pg/ml**	**Mean ±SD**		

**IFN-γ**	2.77 (0.44)	2.78 (0.13)	NA
**TNF-α**	1.10 (0.69)	0.91 (0.51)	NA
**IL-2**	0.45 (1.02)	0 (0)	NA
**IL-8**	54.84 (128.40)	53.66 (110.00)	0.98
**MIP-1 α**	2.47 (0.93)	0.60 (1.01)	NA
**MIP-1 β**	11.77 (9.92)	14.31 (22.29)	0.67
**RANTES**	2.87 (1.82)	4.82 (3.79)	NA

N: number; SD: standard deviation, IFN: interferon, TNF: tumor necrosis factor, IL: interleukin, MIP: macrophage inflammatory protein, RANTES: regulated on activation normal T cell expressed and secreted; NA: not available if the means are below 5 pg/ml.

In contrast, urine IP-10 was detected and found to be increased in the patients with active TB compared to the healthy subjects. Therefore, it was analyzed in all the enrolled individuals using the same methodology (CBA Flex Set) but only evaluating IP-10 (see Material and Methods). Patients with lung diseases were also included.

**Figure 1:**
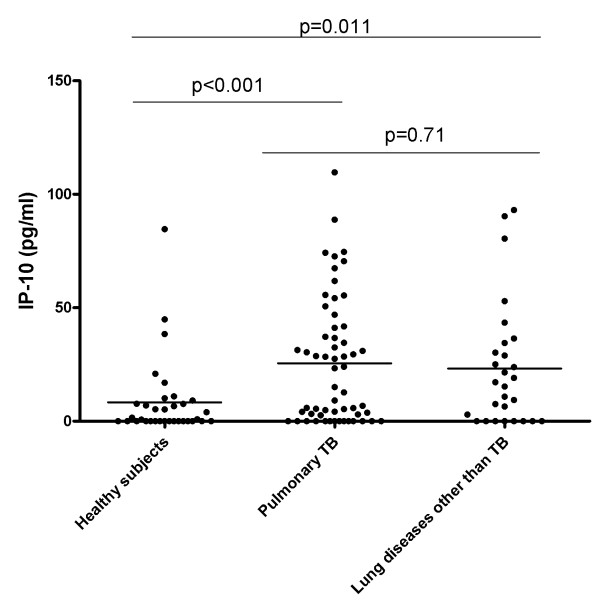
**Urine IP-10 is associated with pulmonary lung diseases**. Urine IP-10 concentrations from healthy subjects, patients with pulmonary TB and "lung diseases other than TB" are reported. Horizontal bars indicate the means. IP-10 was measured by the CBA Flex Set and the data were analyzed by flow cytometry. The statistical significance of the results was evaluated by using the t-test, and differences were considered to be significant when the p-values (indicated by the numbers) were ≤ 0.05.

As shown in Figure 1, urine IP-10 levels in patients with pulmonary TB (mean: 25.54 pg/ml, SD: 27.43) were significantly higher than in the healthy subjects (mean: 8.31 pg/ml, SD: 17.07) (p < 0.001). In the patients with "lung diseases other than TB", the IP-10 level (mean: 23.20 pg/ml, SD: 27.26) was significantly higher than in the healthy subjects (p = 0.011). No significant difference between pulmonary TB patients and patients with "lung diseases other than TB" was observed (p = 0.71).

### Measurement of IP-10 by ELISA

**Figure 2:**
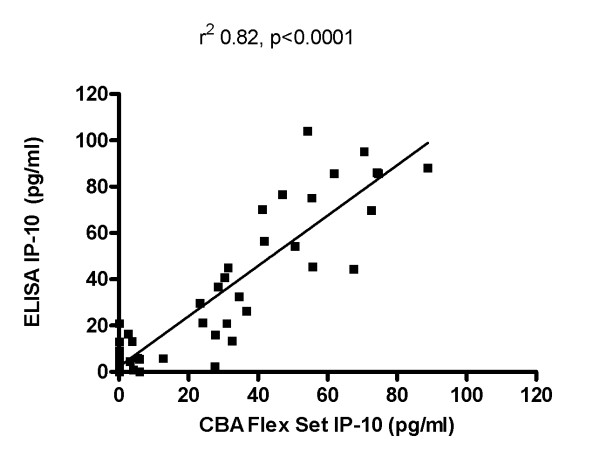
**Positive correlation between the Urine IP-10 levels detected by ELISA and by CBA Flex Set analyzed by flow cytometry**. Urine IP-10 concentrations were evaluated in parallel by ELISA and by CBA Flex set analyzed by flow cytometry. A significantly positive statistical correlation was found between the 2 assays (r^2 ^0.82, p < 0.0001).

Technically, the CBA Flex Set method performs better than routine ELISA because it significantly reduces the quantity of the sample required and the time needed to obtain the final concentration results of one or more of the simultaneously tested immune mediators. However it is an expensive procedure and requires the use of a flow cytometry. Therefore, we assessed whether IP-10 detection could be performed by ELISA with similar accuracy by evaluating the equivalent of these 2 laboratory procedures in 49 samples. As shown in Figure 2, a significantly positive statistical correlation was found between the 2 assays (r^2 ^0.82, p < 0.0001) indicating the feasibility of measuring urine IP-10 levels by ELISA.

### Comparison of IP-10 levels in patients on TB treatment and after completion of therapy

**Figure 3:**
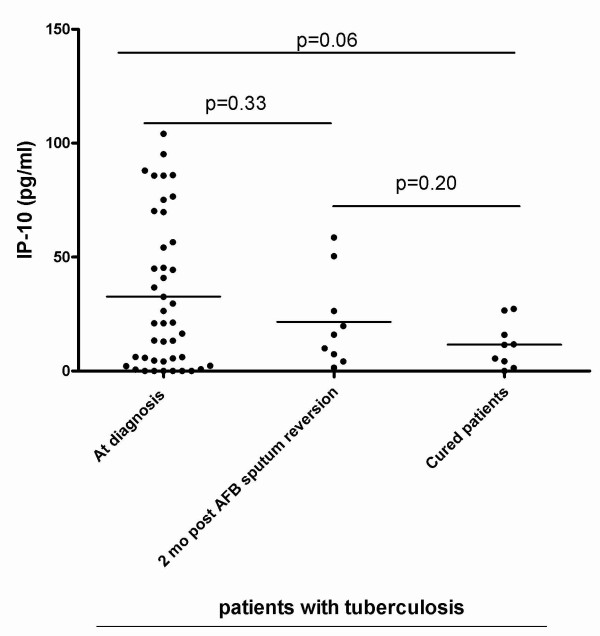
**Overtime evaluation of IP-10 level in patients undergoing TB treatment and after therapy completion**. Urine IP-10 concentration was evaluated by ELISA in patients at the time of TB diagnosis, 2 months after AFB sputum reversion and after therapy completion. Horizontal bars indicate the means. The statistical significance of the results was evaluated by using the t-test. A statistical difference close to significance was found between the IP-10 levels at the time of TB diagnosis and after therapy completion (p = 0.06).

To assess whether IP-10 can be used as a biomarker for monitoring TB treatment, we evaluated the IP-10 level in the urine of 18 patients, 9 of whom were tested 2 months after AFB smear sputum reversion and 9 of whom were cured of TB (Figure 3, Table 1). The IP-10 level was evaluated by ELISA. Compared to patients tested at the time of TB diagnosis (mean 32.69 pg/ml, SD:38.82), the cured TB patients had lower IP-10 levels (mean 11.56 pg/ml, SD:10.12) and this difference was close to statistical significance (p = 0.06). No significant difference was found when patients tested at diagnosis were compared to those tested 2 months after AFB sputum reversion (mean 21.57 pg/ml, SD: 20.32) (p = 0.33). Finally no significant difference was found between the urine IP-10 levels evaluated in cured TB patients and those tested 2 months after AFB sputum reversion (p = 0.20).

## Discussion

In the past, urine neopterin, an immune marker produced by human macrophages [20], has been shown to be a useful parameter for measuring the degree of disease activity and the response to treatment of patients with active TB [26-30]. Differently, in the present proof-of-principle study, for the first time to our knowledge, we analyzed the urine of patients with active TB and lung diseases other than TB for the presence of other immune mediators known to be associated with TB, such as IFN-γ, TNF-α, IL-2, IL-8, MIP-1α, MIP-1β, RANTES and IP-10.

Low levels of IFN-γ, TNF-α, IL-2, IL-8, MIP-1α, MIP-1β and RANTES were found, and no differences were observed between patients with lung diseases and healthy subjects. In contrast, urine IP-10 levels were significantly increased in patients with lung diseases (either TB or non-TB related) compared to healthy subjects in the absence of urinary infections. Moreover TB patients tested at disease onset had higher IP-10 levels compared to patients with cured TB, and this difference was close to statistical significance. IP-10 results obtained by CBA Flex Set were comparable to those obtained by ELISA, rendering the procedure easier to perform and potentially available, even in limited resource settings. Therefore IP-10 is detectable in the urine of patients with lung diseases and this finding may be useful for further clinical and/or research approaches.

If our results could be confirmed in additional, larger studies this would be an important finding, especially given the multiple advantages of collecting urine as opposed to blood.

IP-10 is an important mediator for recruiting activated lymphocytes into the lungs in pulmonary diseases [15,34-36] and is involved in the response to *M. tuberculosis *[37-40] at both the site of TB disease and in peripheral blood. Dheda showed detectable levels of IP-10 in the pleural effusions of TB patients [41], whereas Azzurri described high levels of IP-10 in the plasma of patients with active pulmonary TB, which subsequently decreased after successful anti-TB treatment. These data from the literature may imply that IP-10 is a potential marker for lung diseases and therapy monitoring.

Our results suggest that urine concentrations of IP-10 may have the same course during TB treatment as that recorded in plasma, and thus propose that urine levels of this chemokine should be evaluated as a potential biomarker for monitoring TB treatment.

Regarding the presence of IP-10 in urine, previous studies have shown its detection only in kidney-related diseases. In particular, higher urine IP-10 concentrations were found during urosepsis and in those with febrile urinary tract infections compared to healthy subjects, accompanied by a significant decrease following successful therapy [42,43]. Interestingly, IP-10 concentrations were also increased in human urine upon experimentally induced endotoxemia [43]. Furthermore, reports have shown an increase of mRNA IP-10 levels in the urine of patients with autoimmune diseases, such as class IV of lupus nephritis, when compared to other classes of lupus nephritis [44].

We also adjusted the IP-10 concentrations in urine with creatinuria and with the urine specific gravity in order to exclude any dilution factor as responsible for the results obtained, as previously shown [30]. However, significant differences were still found between the ratios of the healthy subjects compared to both groups of patients, with pulmonary TB or with lung diseases other than TB, even after these adjustments.

The present study has a number of limitations. The evaluation of IP-10 as a marker for therapy efficacy was performed only in patients with TB, excluding those with lung diseases other than TB. The comparison between patients with active TB, on treatment and after completion of therapy was performed by cross-sectional evaluation, not using a formal longitudinal study. However, despite these limitations, the study shows a certain consistency of the data obtained among all the patients with lung disease in terms of higher detection of IP-10 compared to the healthy subjects. Moreover, although of a cross-sectional design, the study shows a strong trend for lower concentrations of IP-10 in urine following successful TB treatment. Although a moderate number of patients were enrolled (138), certainly larger studies are needed to evaluate the robustness of these results.

## Conclusions

For the first time, IP-10 (in contrast to IFN-γ, TNF-α, IL-2, IL-8, MIP-1α, MIP-1β and RANTES) has been detected in the urine of patients with lung disease in the absence of renal illnesses. In those with active TB, its level may decrease after completion of TB therapy. Further studies are needed to substantiate our findings.

## List of abbreviations

ABF: acid fast bacilli; BCG: bacillus Calmette-Guérin; CBA: cytometric bead array; COPD: chronic obstructive pulmonary disease; EDTA: ethylenediaminetetraacetic acid; ELISA: Enzyme Linked ImmunoSorbent Assay; HIV: human immunodeficiency virus; IFN: interferon; IL: interleukin; INMI: National Institute for Infectious Diseases; IP: Interferon-γ-inducible protein; M: Mycobacterium; MIP: macrophage inflammatory protein; NA: not available; RANTES: regulated upon activation normal T cell expressed and secreted; SD: standard deviation; TB: Tuberculosis; TNF: tumor necrosis factor; TRIS HCl: Tris(hydroxymethyl)aminomethane hydrochloride; UTI: urinary tract infections.

## Competing interests

The authors declare that they have no competing interests.

## Authors' contributions

AC and LC carried out the experimental data, the data analysis and drafted the manuscript; TC and VV carried out the experimental data and drafted the manuscript; EG and DG were responsible for the statistical analysis; GC and DG were responsible for patient selection and enrolment; GC helped in the data analysis; DG was responsible for the conception of the study design, the organization of the people involved in the study, the data analysis and the preparation of the manuscript. All authors read and approved the final manuscript.

## Pre-publication history

The pre-publication history for this paper can be accessed here:

http://www.biomedcentral.com/1471-2334/10/333/prepub
